# On Lienteric Diarrhœa in Children

**Published:** 1873-01

**Authors:** Eustace Smith


### ON LIENTERIC DIARRHŒA IN CHILDREN.

BY EUSTACE SMITH, M.D.

A variety of diarrhœa is not uncommon in children, the peculiarity of which consists in the fact that the motions contain little fæcal matter, but are composed almost entirely of undigested food mixed with mucus so as to present a slimy appearance. These motions are passed very shortly after, or even during a meal; the food taken appears to pass with extraordinary rapidity along the alimentary canal, and to be voided in almost the same state in which it was swallowed.

The condition which gives rise to this looseness of the bowels is no doubt an unnatural briskness of peristaltic action; the intestines are in a state of great irritability, so that food taken into the stomach is at once forced along the digestive tract with a rapidity which allows little digestion to take place during its passage.

This form of diarrhœa is found in children of from three or four to nine or ten years of age, and gives rise to much emaciation, pallor, languor, and to all the other signs of defective nutrition. At the same time the other consequences of irritable bowels are usually to be noted, and in bad cases night-terrors, nocturnal incontinence of urine, sudden faintness, unusual fretfulness and causeless crying are commonly sources of great anxiety to parents, who attribute them at once to that great bugbear of mothers, "irritation of the brain."

The bowels act three, four or more times during the course of the day. There is almost always an evacuation in the morning on first rising from bed, and afterwards in the course of the day each meal is at once followed by a like movement of the bowels, the child having often to leave the table hurriedly, and frequently before the repast is actually concluded. Each motion is preceded by griping pains in the belly, and characterized by excessive urgency, the patient having great difficulty in restraining his desire during the time necessary to enable him to reach the closet. These griping pains are not always followed by a stool, but may come on and go off at irregular times in the course of the day without any result. Sometimes, however, they are accompanied by a desire to go to stool, although no motion is actually passed. The tongue may be a little furred, but is usually clean, and is red at the tip and edges; the redness being due to small crimson papillæ, which are sometimes slightly elevated.

This variety of diarrhœa is not to be controlled by the ordinary astringents, and is very much increased by aperients, as castor-oil. Opium will, however, often check it temporarily; but for its permanent cure, the best remedies are arsenic and tincture of nux vomica in small doses. The abnormal activity of peristaltic action to which the derangement is owing, appears to be due to impressions of cold; and, therefore, that the cure may be permanent, it is advisable to protect the belly by a broad flannel belt, so that the body may not be exposed to sudden chills.

The following short cases will serve as illustrations of this form of diarrhœa, and of the treatment to which it is most readily amenable:

Master R., aged five years, was brought up to me from the country. "For the last twelve months he has been subject to prolonged attacks of diarrhœa, during which the bowels will act four or six times in the day. The motions usually follow a meal, and sometimes so urgently that he is obliged to leave the table before the repast is concluded. The motions are said 'to run from him,' and to contain much slimy matter. There is no straining, but the stools are preceded by some griping pains in the belly. He is fretful and rather thirsty, but sleeps well at night. He is losing flesh rapidly, although the appetite is large." The child was ordered to take three drops of tincture of nux vomica in a draught of citrate of potash three times a day before meals. A few days afterwards he returned much better. The bowels were acting three times a day, not after meals. His appetite was more easily satisfied, and he had ceased to waste. A change was then made in his medicine to one drop of liq. arsenici chlor. in a nitric acid mixture before each meal, and he was not seen again.

Elizabeth W., aged four years, had suffered for a long time from repeated attacks of diarrhœa, each of which lasted for many weeks together. The motions were said to follow immediately upon taking food, and to occur sometimes in the intervals between the meals, so that the bowels were frequently acted upon five or six times in the day. The stools were slimy, and were passed without straining or apparent discomfort. The child was irritable in temper, and was very restless at night. Her appetite was capricious, and she was very fanciful in her eating. Her skin was exceedingly rough and dry. The tongue was clean, and over the surface were scattered light-red elevated papillæ. She was first seen on January 7, and was ordered to take one drop of liq. arsenicalis in a mixture containing citrate of iron and ammonia, with bicarbonate of soda, three times a day. A warm bath was recommended every night, for the purpose of softening the skin and removing the dry epithelium scales.

On January 14 the girl seemed better in herself, although the bowels were in the same condition as before. She had taken the medicine regularly, but each meal was followed by the accustomed stool, and the child was still losing flesh. A mixture was then ordered containing laudanum and tincture of nux vomica—two drops of each to be taken before each meal. Considerable improvement followed the change of medicine—the appetite became very good, and the bowels, although still relaxed after each meal, yet did not act in the intervals, so that the daily number of evacuations was much reduced. The skin, owing to the nightly warm bath, had become soft and supple. One drop of liq. arsenici chlor. was then given three times a day in nitric acid mixture, and the cure was soon complete.

In the following case, the lienteric diarrhœa was associated with incontinence of urine: Emma L., a girl eight years old, who had suffered from rickets during infancy, was brought for advice for an almost constant looseness of the bowels. The stools occurred directly after meals. She also complained of an inability to hold her water for long together, either in the day or night. She was ordered three drops of liq. strychniæ and ten drops of tincture of belladonna three times a day before meals. A few days afterwards she could hold her water very much better, but the bowels were still relaxed after meals, and were as loose as ever. One drop of liq. arsenici chlor. was then given with dilute nitric acid three times a day before meals, and in the course of a week greatly improved the character of the stools, which were more solid and healthy-looking, and were less commonly found to follow a meal, although they were still too frequently passed. The girl was said to be very nervous, and to complain of slight pains in the belly. The mixture was then changed to—

℞.—Tinct. nucis vomicæ, ♏v.; liq. arsenicalis, ♏j.; mist. pot. citrat., ℥ ss., three times a day; and the motions soon became perfectly natural, both in number and quality.—*Medical Times and Gazette, June,* 1872.

